# Nuclear Transport: A Switch for the Oxidative Stress—Signaling Circuit?

**DOI:** 10.1155/2012/208650

**Published:** 2011-10-15

**Authors:** Mohamed Kodiha, Ursula Stochaj

**Affiliations:** Department of Physiology, McGill University, Montreal, QC, Canada H3G 1Y6

## Abstract

Imbalances in the formation and clearance of reactive oxygen species (ROS) can lead to oxidative stress and subsequent changes that affect all aspects of physiology. To limit and repair the damage generated by ROS, cells have developed a multitude of responses. A hallmark of these responses is the activation of signaling pathways that modulate the function of downstream targets in different cellular locations. To this end, critical steps of the stress response that occur in the nucleus and cytoplasm have to be coordinated, which makes the proper communication between both compartments mandatory. Here, we discuss the interdependence of ROS-mediated signaling and the transport of macromolecules across the nuclear envelope. We highlight examples of oxidant-dependent nuclear trafficking and describe the impact of oxidative stress on the transport apparatus. Our paper concludes by proposing a cellular circuit of ROS-induced signaling, nuclear transport and repair.

## 1. Introduction

### 1.1. Reactive Oxygen Species

Oxidative stress is generated by an increase in reactive oxygen species (ROS), either in the form of free radicals or nonradical oxidants [1, 2]. Although elevated levels of ROS can damage a wide variety of molecules, ROS production is essential to normal cell physiology [3–12]. As such, ROS participate in cell-signaling events and can function as second messengers. Moreover, ROS are generated at sites of inflammation, where they fend off microbial infections [13–16]. On the other hand, ROS are believed to contribute to aging [3–9, 12]; they are also produced in response to environmental insults, such as X-rays, UV light, ultrasound, or microwave radiation [17–19]. At the cellular level, ROS are generated as metabolic byproducts of normal biological processes, with oxidative phosphorylation in mitochondria as the primary source in eukaryotic cells [20]. Aside from the mitochondrial electron transport chain, NADPH oxidases, cyclooxygenases, lipoxygenases, xanthine oxidase, and other cellular enzymes make also important contributions to cellular ROS production [21–25].

The different types of ROS and their mode of action have been discussed in detail [1, 11, 26–30]. ROS that are particularly important to cell physiology include the hydroxyl radical •OH, superoxide anion •O_2_^−^, the nonradical hydrogen peroxide (H_2_O_2_), alkoxy and peroxy radicals, hypochlorous acid or peroxynitrite, and reactive sulfur species [1, 29, 31, 32]. Here, we recapitulate the properties of those ROS only that are relevant to the experiments discussed in this review.

The hydroxyl radical •OH is highly reactive and causes damage to nucleic acids and proteins, this radical also promotes lipid peroxidation [2, 12, 33]. Due to their high reactivity, hydroxyl radicals are especially harmful and considered a major cause of oxidant-induced damage [34]. The superoxide free radical •O_2_^−^ can interfere with the proper function of enzymes by damaging their active sites, with cysteine residues being particularly susceptible [32]. In an experimental setting, superoxide radicals can be generated by providing xanthine oxidase with the appropriate substrates [35].

There is some debate about the impact of H_2_O_2_ on the cellular redox homeostasis. On one hand, H_2_O_2_ is not deemed a major direct threat for the cellular redox homeostasis due to its poor reactivity towards biomolecules [36]. However, H_2_O_2_ rapidly translocates through lipid bilayers and is a potential precursor for •OH radicals [32, 37]. Thus, high concentrations of H_2_O_2_ can release iron from heme proteins and catalyze the conversion of H_2_O_2_ to hydroxyl radicals [37]. It was also proposed that the nonradical oxidant H_2_O_2_ may have profound effects on redox signaling in living cells, where it alters the function of redox circuits that are composed of redox-sensitive building blocks [1]. Despite these different views on how H_2_O_2_ contributes to oxidant-induced damage, we and others [38–42] have used this compound extensively to examine the impact of oxidative stress on nuclear transport (see below).

### 1.2. Oxidative Stress and Cellular Defense Mechanisms

The appropriate response to stress is fundamental to cell survival and the recovery from disease-related or environmental damage [3, 5, 6, 9, 11]. Thus, in order to maintain redox homeostasis, the balance between production and clearance of ROS is essential. Imbalances in ROS concentration, if left without proper intervention, can interfere with a wide variety of cellular processes, leading to serious injuries and possibly cell death, either by apoptosis or necrosis [28, 43].

Upon accumulation, ROS can interact inappropriately with a large number of biomolecules, including lipids, proteins, and DNA, thereby interfering with numerous cellular functions [28, 37]. For instance, ROS may induce damage to various enzymes, leading to the partial or complete loss of their function. Notably, ROS-damaged proteins can form toxic aggregates that cause cell injury and ultimately cell death [16]. Furthermore, ROS-induced lipid peroxidation may alter the permeability of cellular membranes, potentially destroying the membrane integrity and triggering cell death [33, 44]. In addition, ROS-induced modifications of DNA can be mutagenic, possibly initiating cell transformation and promoting cancer [45].

In line with the complex pattern of damage triggered by oxidative stress, ROS accumulation contributes to the pathophysiologies of many human diseases and syndromes. In particular, oxidative stress plays a critical role in the onset and the progression of neurodegenerative disorders, diabetes, cardiovascular diseases, and nephropathy [27, 46–58].

To counteract the potential damage of elevated ROS concentrations, cells have developed different strategies that limit the action of reactive compounds and prevent their accumulation. To this end, eukaryotic cells are equipped with multiple defense mechanisms that promote the removal and inactivation of ROS in different cellular compartments [59–62]. These mechanisms rely on the coordinated action of several enzymatic systems that are able to react with and neutralize different ROS. For example, the superoxide dismutase (SOD) system is essential to redox homeostasis [11, 63–65], as it catalyzes the conversion of •O_2_^−^ to H_2_O_2_. H_2_O_2_ produced by SOD can then be eliminated by the enzymatic action of catalases.

The glutathione/glutathione disulfide system (GSH/GSSG) is one of the major contributors to redox homeostasis and of particular importance to the intracellular redox state. Accordingly, glutathione is believed to be the primary defense when cells are injured by oxidative stress during ischemia/reperfusion [66, 67]. Moreover, changes in the GSH/GSSG ratio affect the intracellular redox state, and depletion of intracellular glutathione generates oxidative stress [61]. Owing to its pivotal importance to redox homeostasis, imbalance of the GSH/GSSG system has been linked to many human diseases, pathologies, and aging [11, 66, 68]. The GSH/GSSG system can be modulated experimentally, and diethyl maleate is one of compounds that deplete glutathione, thereby causing oxidative stress [38, 69]. Furthermore, the cellular redox homeostasis can also be altered by changing the activity of glutathione peroxidase, glutathione, or thioredoxin reductase.

## 2. Oxidative Stress and Nucleocytoplasmic Transport

### 2.1. Nuclear Transport of Macromolecules

**Figure 1:**
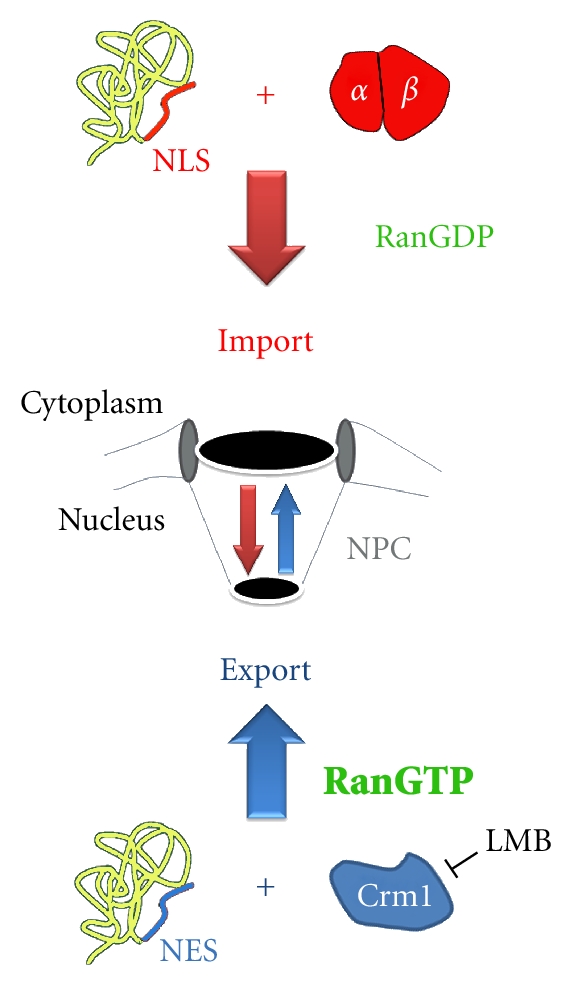
Simplified model for classical nuclear import and Crm1-mediated export, two essential transport pathways. Classical nuclear import depends on the carrier importin-*β* and the adaptor protein importin-*α*. Together, importin-*α*/*β* move NLS-containing cargos to the nucleus. The absence of RanGTP from the cytoplasm permits the assembly of import complexes in the cytoplasm. Conversely, the high RanGTP concentration in the nucleus promotes the dissociation of classical import complexes after they translocate across the NPC. RanGTP in the nucleus is also necessary to generate export complexes that contain Crm1 and NES-containing cargo. The function of Crm1 is inhibited by leptomycin B (LMB).

Nucleocytoplasmic transport is central to the cellular homeostasis, as the proper and timely response to endogenous and environmental stimuli relies on the communication between the nucleus and cytoplasm. This applies in particular to kinases and phosphatases, many of which move in and out of the nucleus in response to oxidants or other stressors (see below). The nuclear envelope provides the barrier between these two compartments [70, 71], and macromolecules traverse the nuclear envelope via nuclear pore complexes (NPCs). Trafficking in and out of the nucleus controls signal transduction, gene expression, cell-cycle progression, and apoptosis; regulated nuclear transport is also essential for development and required for the proper response to stress [72–75]. The separation of nucleus and cytoplasm is ideal to divide signaling and other events. However, this compartmentalization can impede the intracellular communication if components of the nuclear transport apparatus are affected by ROS. This is indeed the case, as nuclear transport factors are primary cellular targets for oxidants. Before describing the impact of oxidative stress on nuclear transport, we briefly summarize those mechanisms of nuclear trafficking that are relevant to our review (Figure 1).

Although diffusion across the NPC is not simply a function of the molecular mass, most proteins that are larger than 40 kD do not efficiently diffuse across the nuclear envelope. Nevertheless, molecules exceeding the diffusion channel of the NPC can move in or out of the nucleus if they carry specialized transport signals. Nuclear localization (NLS), nuclear export (NES), or shuttling sequences serve as permanent signals that mediate targeting to the proper location. Classical NLSs are characterized by clusters of basic amino acid residues, whereas NESs are frequently enriched for leucine or isoleucine residues. However, the final destination of a macromolecule not only depends on such transport signals; the steady-state distribution is also controlled by its retention in the nuclear or cytoplasmic compartment.

**Nuclear Carriers**

**Figure 2:**
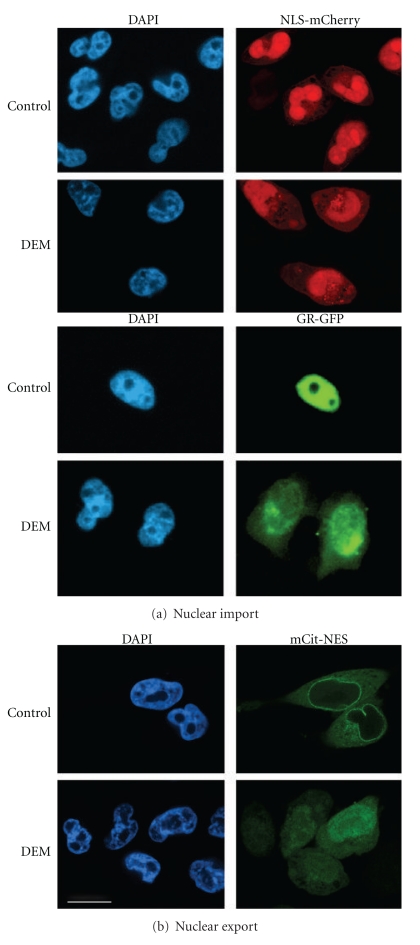
Oxidative stress inhibits classical nuclear import and Crm1-mediated export. (a) Nuclear import. HeLa cells transiently synthesizing the import substrates NLS-mCherry or GFP-tagged glucocorticoid receptor (GR-GFP) were incubated under nonstress conditions (control) or with DEM as described [69]. Note that a significant amount of the reporter proteins relocated to the cytoplasm upon oxidant treatment, indicating that classical nuclear import was inhibited. (b) Nuclear export. HeLa cells synthesizing the fluorescent reporter protein mCit-NES, a Crm1 cargo, were exposed to DEM and processed as in part a. The Crm1 export cargo was excluded from the nucleus under control conditions, but relocated to nuclei upon oxidative stress. Size bar is 20 *μ*m.

Nuclear transport of most proteins depends on transporters of the importin-*β* group (also called karyopherin-*β*). Importin-*β* family members interact with their cargo either directly or through an adaptor. The latter applies to classical nuclear import, which relies on the carrier importin-*β*1 and its adaptor importin-*α* (Figure 1). Multiple isoforms of importin-*α* exist in higher eukaryotes, where they recognize classical NLSs in endogenous and fluorescent cargos such as NLS-mCherry (Figure 2(a)). Crm1/exportin-1 [76], another importin-*β* family member, moves NES-containing proteins like mCit-NES to the cytoplasm (Figure 2(b)). This transport route can be inhibited specifically with leptomycin B, a compound that covalently modifies a cysteine residue of Crm1 [77].

**The RanGTPase System**

Carriers of the importin-*β* family require the small GTPase Ran and factors that modulate Ran activity. These factors include in the cytoplasm RanBP1 (Ran-binding protein 1) and the GTPase activating protein RanGAP1, with RanGAP1 binding to Nup358 at the cytoplasmic side of the NPC. By contrast, the RanGTP-binding protein RanBP3 and the guanine nucleotide exchange factor RCC1 (RanGEF) are located in the nucleus, where RCC1 binds to chromatin. The asymmetric distribution of Ran modulators generates a gradient across the nuclear envelope, with RanGTP in nuclei and RanGDP in the cytoplasm (Figure 1). This gradient provides the driving force for all importin-*β* dependent transport [70, 71].

**Regulation of Nuclear Transport**

Control of nuclear trafficking is crucial under normal, stress, and disease conditions, and it occurs on multiple levels [72, 73]. For instance, phosphorylation and other posttranslational modifications can change the transport of individual cargos [73, 78]. A more general regulation that affects multiple transport cargos is achieved by targeting components of the nuclear transport machinery. This can be accomplished by altering the localization or posttranslational modification of transport factors, and such changes are observed in response to oxidative stress [72].

The following sections summarize the effects of oxidative stress on specific cargos that are relevant to human health, the nucleocytoplasmic transport apparatus, and important signaling components. We will then build on this information to propose that the interdependence of oxidative stress, nucleocytoplasmic transport, and signaling provides a circuit that controls cell survival.

### 2.2. Oxidative Stress Impinges on Multiple Nuclear Cargos

As discussed above, oxidative stress causes the modification of targets in the nucleus and cytoplasm. Together, ROS-dependent modifications of cargos and the nuclear transport apparatus regulate the intracellular distribution of many of these targets. Among the oxidant-sensitive targets that translocate through NPCs are transcription factors, some of which are also implicated in the stress response. Prominent examples of transcription factors that relocate in response to oxidative stress are NF-*κ*B and Nrf2 (NF-E2-related factor 2). The ROS-mediated redistribution of NF-*κ*B and Nrf2 has been described extensively [80–83] and the relevant data will only be summarized here. Our discussion will focus on high-mobility group box 1 protein (HMGB1) and glycerolaldehyde-3-phosphate dehydrogenase (GAPDH) to illustrate the link between ROS, nuclear trafficking and signalling.

The role of NF-*κ*B in immunity and inflammation is well established; however, this transcription factor is also critical for the synthesis of antioxidant proteins [80, 81]. The genes upregulated by NF-*κ*B include MnSOD, Cu,ZnSOD, and HO-1 (heme oxygenase 1), all of which participate in antioxidant defense processes. ROS and numerous other stimuli control the intracellular distribution of NF-*κ*B. In the absence of these stimuli, NF-*κ*B is retained in the cytoplasm due to its association with I-*κ*B. ROS trigger the degradation of I-*κ*B, thereby promoting the nuclear accumulation of NF-*κ*B and the subsequent transcription of genes that contain NF-*κ*B response elements [81].

Nrf2 is another key player in the antioxidant response that relocates upon oxidant exposure. Under nonstress conditions, concentrations of the transcription factor Nrf2 are low, and the protein is retained in the cytoplasm owing to its association with Keap1 [82, 83]. In response to oxidative stress, a complex series of events leads to the stabilization of Nrf2 and its translocation into the nucleus. In the nucleus, Nrf2 upregulates the expression of several genes that are implicated in the antioxidant response [84]. The oxidant-induced nuclear accumulation of Nrf2 can be mediated by importin-*α*5/importin-*β*1 [85], whereas Nrf2 nuclear export is promoted by Crm1 [86]. Phosphorylation of Nrf2 likely plays a role in its nuclear import and export, with PI3 kinase possibly stimulating Nrf2 nuclear accumulation [83, 84].

More recent studies identified HMGB1 and GAPDH as redox sensitive proteins whose nucleocytoplasmic distribution is regulated by ROS and signaling [87, 88]. Like Keap1/Nrf2, HMGB1 functions as a redox sensor [87]. In nuclei, HMGB1 serves as a DNA chaperone and participates in replication, transcription, as well as DNA repair. However, HMGB1 also contributes to a variety of signaling processes, which involve HMGB1 export to the cytoplasm and its subsequent secretion. At steady-state HMGB1 shuttles between the nucleus and cytoplasm, but hyperacetylation triggers its relocation to the cytosol [89]. It was speculated that lysine acetylation reduces the number of positive charges and thus interferes with nuclear import of the protein [89]. Karyopherin-*α*1, a member of the importin-*α* family, was identified as a binding partner that supports *in vitro* nuclear import of HMGB1, most likely in conjunction with importin-*β*1 [90]. The interaction of HMGB1 with karyopherin-*α*1 can be abrogated by phosphorylation, and modification of two NLS segments is necessary to relocate HMGB1 to the cytoplasm [90]. Taken together, a combination of acetylation and phosphorylation controls HMBG1 nuclear accumulation. These posttranslational modifications likely prevent the recognition of HMBG1 by the classical import apparatus.

Nuclear export of HMGB1 is at least in part mediated by Crm1, as leptomycin B drastically reduced HMGB1 exit from the nucleus [89]. Treatment with H_2_O_2_ upregulated the interaction Crm1/HMBG1 and relocated HMBG1 to the cytoplasm for secretion [91]. This oxidant-dependent secretion was sensitive to JNK and MEK inhibitors, in line with the idea that several members of the MAP kinase families control HMBG1 movement from the nucleus to the cytoplasm and its subsequent release. In other studies, IL-1*β*-dependent ERK1/2 activation increased the concentration of Crm1 and led to HMBG1 accumulation in the cytoplasm [92]. Whether H_2_O_2_ treatment, which activates ERK1/2, has the same effect on Crm1 levels is an exciting question that has to be answered in the future.

In recent years GAPDH has emerged as an enzyme that is involved in diverse cellular processes [88, 93]. Thus, GAPDH not only functions in glycolysis in the cytoplasm, but also plays additional important roles in other compartments of the cell, including the nucleus [88, 94–101]. The nuclear accumulation of GAPDH is controlled by posttranslational modifications and the interaction with different binding partners in the cytoplasm and nucleus. In response to oxidative stress, GAPDH undergoes S-nitrosylation and subsequent association with Siah. The GAPDH-Siah complex then moves into the nucleus, where it participates in the regulation of gene expression and apoptosis [88]. GAPDH nuclear accumulation depends on the acetylation of three lysine residues by the acetyltransferase p300 [101]. Furthermore, *O*-GlcNAc glycosylation of GAPDH occurs close to the Siah-binding site, and this modification promotes GAPDH nuclear accumulation [100]. Although not tested by the authors, *O*-GlcNAc modifications rise in response to oxidative stress [102] and could therefore assist in the stress-induced nuclear accumulation of GAPDH. Interestingly, the nucleocytoplasmic trafficking of GAPDH has been linked to several signaling pathways. In particular, activation of AMPK promoted the nuclear accumulation of GAPDH, whereas signaling through the PI3 kinase → Akt module is required for Crm1-dependent nuclear export [96].

The intracellular location of GAPDH is directly relevant to human health (see below). For example, when in the nucleus GAPDH might contribute to the initiation of apoptosis in brain cells. Moreover, the oxidant-induced changes in GAPDH subcellular localization probably play a role in the pathology of Alzheimer disease [93]. GAPDH is also critical to the development of diabetic complications, and changes in its nuclear accumulation might aggravate diabetic retinopathy [97].

Taken together, there is a growing list of proteins whose nucleocytoplasmic distribution is controlled by the intracellular redox homeostasis. This regulation frequently relies on posttranslational modifications, which can alter the interaction of a particular cargo with its carrier or the retention in nuclear and cytoplasmic compartments.

### 2.3. Oxidative Stress as a Key Player in Human Health

The cellular damage caused by oxidative stress promotes the onset as well as progression of several diseases and pathophysiologies. Thus, oxidative stress plays a critical role in neurodegenerative disorders, cardiovascular and metabolic diseases, as well as the complications associated with diabetes. Here, we focus on some examples that highlight the adverse effects of oxidative stress on human health.

**Oxidative Stress and Neurodegenerative Diseases**

The human brain is particularly vulnerable to oxidant-induced damage owing to high oxygen consumption, lipids rich in polyunsaturated fatty acids, high amounts of redox-active transition metals, and relatively poor defense against oxidative stress [30, 48, 103]. Several lines of evidence implicate oxidative stress in the neuronal damage that accompanies neurodegenerative disorders [25, 30, 34, 103, 104]. For instance, analysis of cerebrospinal fluid, plasma, and urine samples or postmortem brain specimens demonstrated the increase in oxidative damage in patients suffering from amyotrophic lateral sclerosis [105], Friedreich ataxia, Parkinson, Alzheimer, and Huntington diseases [30, 48, 103]. Oxidant-induced injury is elevated in the brain at early stages of these diseases, supporting the model that oxidative stress contributes to the etiology of neurodegeneration. In line with this hypothesis, mitochondrial dysfunction and oxidative damage to mitochondrial proteins are shared features of different neurodegenerative diseases [25, 30, 34, 103]. Animal models further support this idea, as inhibitors of mitochondrial function can induce some of the pathologies associated with Parkinson disease [34]. In addition, proteomics identified a large number of proteins that show increased oxidative damage in patients suffering from various forms of neurodegeneration. These proteins include several enzymes that are critical to oxidative phosphorylation and glycolysis. Notably, when compared to control subjects GAPDH oxidation was increased in Alzheimer and Parkinson patients; GAPDH was also affected in ALS mouse models [103]. This is significant, because GAPDH and its subcellular trafficking are of particular importance to human metabolism and the pathologies associated with neurodegenerative diseases. As such, oxidative damage not only reduces the enzymatic activity of GAPDH in Alzheimer disease, but also supports the association with Siah and the subsequent translocation of the GAPDH-Siah complex to the nucleus (see above). In Alzheimer disease, both GAPDH expression and nitrosylation are increased, probably leading to elevated concentrations of GAPDH-Siah in the nucleus, which in turn promotes apoptosis [93]. Taken together, the oxidant-induced changes in GAPDH enzyme activity and intracellular distribution will reduce the energy supply and advance apoptosis in the brain of Alzheimer patients. Since GAPDH is an established target of oxidative damage in several neurodegenerative diseases [103], it is possible that its oxidant-dependent change in nuclear transport and the subsequent increase in cell death are common to multiple forms of neurodegeneration. Interestingly, GAPDH also plays a critical role in the development of diabetic complications.

**Oxidative Stress and Diabetes**

Oxidative stress is crucial to the etiology of diabetes mellitus and the ensuing damage to different tissues and organs [27, 49, 55, 106, 107]. Thus, oxidative stress can alter insulin signaling by targeting insulin receptor and insulin receptor substrates or through the activation of ser/thr kinases that regulate insulin signaling [55]. In this scenario, the ROS-induced changes to the insulin signaling pathway will advance insulin resistance and the subsequent development of diabetes. PI3 kinase and the MAP kinases ERK1/2 are major components of insulin-mediated signaling. Interestingly, signaling through these kinases is also modulated by oxidative stress and regulates nuclear trafficking (see below).

Oxidative stress not only promotes the development of diabetes, but diabetes also triggers the increase in oxidative stress due to elevated blood glucose and free fatty acids. Such disease-induced ROS production further exacerbates cellular damage and contributes to diabetic complications. In the following, we discuss some of the routes that generate oxidative stress in the diabetic patient [49, 55, 106–108].

Hyperglycemia rises intracellular glucose concentrations and the subsequent production of pyruvate, which is ultimately metabolized via the tricarboxylic acid cycle. As a result of the high abundance of pyruvate, increased amounts of NADH and FADH_2_ are generated by the tricarboxylic acid cycle. Both NADH and FADH_2_ enter into the mitochondrial electron transport chain, but their excess interferes eventually with the proper transfer of electrons. As a consequence of this overload, superoxide production by mitochondria increases and promotes cellular damage, especially in the diabetic vasculature [109, 110]. The importance of mitochondria in hyperglycemia-induced injuries was demonstrated experimentally, as inhibitors of the electron transport chain, upregulation of the uncoupling protein UCP1, or mitochondrial SOD ameliorated some of the damage [49, 106].

The excess of mitochondrial superoxide, combined with other hyperglycemia-induced changes, culminates in secondary diabetic complications. In particular, diabetic nephropathy, retinopathy, neuropathy, and cardiomyopathy arise from the modulation of multiple biochemical pathways, some of which alter the cellular redox homeostasis [27, 49, 107]. For example, upon diabetes, the abundance of intracellular glucose and glycolytic metabolites leads to the increased production of sorbitol and other sugar alcohols by the polyol pathway. This generation of sugar alcohols mediated by members of the aldo-keto reductase family relies on the conversion of NADPH to NADP^+^ [49]. Since NADPH is necessary to generate GSH from GSSG, excessive NADPH consumption will compromise the antioxidant defense and promote ROS-induced damage.

Moreover, ROS concentrations can also be elevated by hyperglycemia-dependent changes in cell signaling. As described above, GAPDH is sensitive to oxidative stress, and the inhibition of GAPDH by ROS increases intracellular concentrations of triose phosphate, a precursor of the PKC activator diacylglycerol. Hence, hyperglycemia triggers PKC activation, thereby changing the signaling events in the diabetic retina, heart, and endothelial cells [49, 106]. Moreover, this hyperglycemia-induced PKC activation is particularly detrimental to the kidney, as it stimulates ROS production by NAD(P)H oxidases and advances diabetic nephropathy [106, 111]. 

Like other forms of stress, diabetes modulates the nucleocytoplasmic distribution of transcription factors, with NF-*κ*B as a prominent example [112]. Similarly, high glucose concentrations accumulated GAPDH in the nucleus of bovine retinal endothelial cells [97], where it could contribute to the progression of diabetic retinopathy.

The downstream effects of hyperglycemia further include changes in the posttranslational modification of proteins. Thus, elevated glucose concentrations raise the amount of fructose-6-phosphate that enters the hexosamine pathway [27, 106], which in turn increases the production of UDP-*N*-Acetylglucosamine and the subsequent *O-*GlcNAc modification of proteins. These changes are important to nuclear transport, because nucleoporins are well established targets for *O*-GlcNAc-glycosylation.

In summary, oxidative stress is implicated in different pathophysiological conditions, some of which alter the proper coordination of nuclear and cytoplasmic events. As discussed in the following section, ROS impinge on the nuclear transport apparatus and thereby modify the communication between nucleus and cytoplasm.

### 2.4. Nuclear Transport and Redox Homeostasis

**Figure 3:**
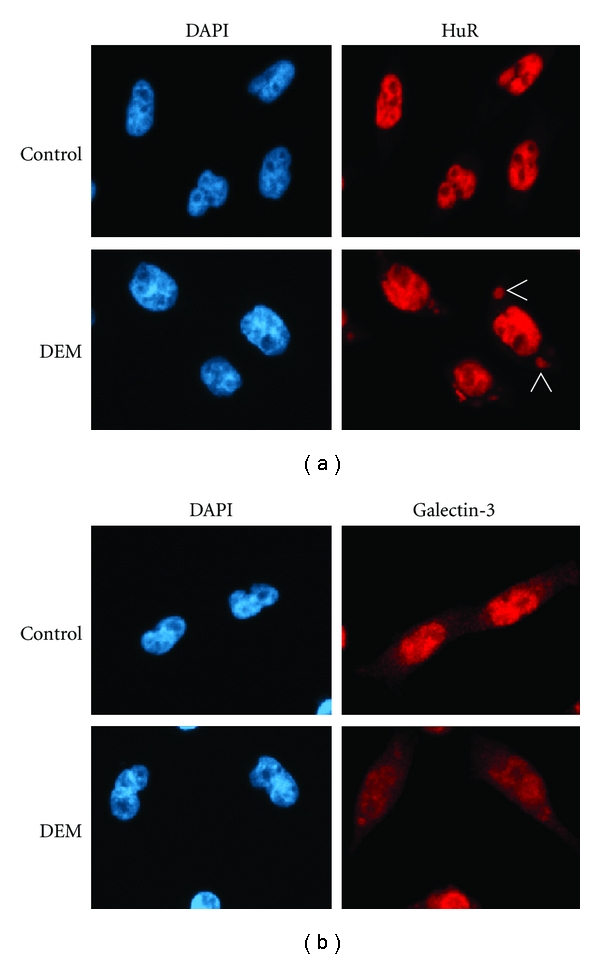
Oxidative stress interferes with importin-*α*1-dependent import of endogenous cargos. The import of two endogenous proteins, the RNA-binding protein HuR and galectin-3, was monitored in HeLa cells under the conditions described for Figure 2(a). Importin-*α*1 promotes nuclear import of both proteins. HuR and galectin-3 were visualized by indirect immunofluorescence and nuclei were stained with DAPI [69]. HuR was nuclear in control cells and redistributed to the cytoplasm of stressed cells, where it accumulated in stress granules (SGs). Similarly, galectin-3 was more concentrated in the nuclei of control cells and relocated to the cytoplasm upon DEM treatment. Arrows indicate the position of some of the SGs.

Changes in cell physiology affect nucleocytoplasmic trafficking in a wide variety of eukaryotes, and the effects of oxidative stress on the nuclear transport apparatus have been analyzed during the past years. We have shown for the yeast *S. cerevisiae* and mammalian culture cells that different forms of stress, including oxidants, heat, and nutrient deprivation inhibit classical nuclear import and export [38, 39, 69, 72, 79, 113–121]. Our previous studies examined the impact of severe and mild oxidative stress. While severe oxidative stress was produced with high concentrations of H_2_O_2_ [39], mild oxidative stress was generated by the oxidant diethyl maleate, DEM [69]. Under severe stress conditions, cells underwent apoptosis, but a large fraction of cells survived the milder stress inflicted with DEM [69]. Nevertheless, Figures 2 and 3 show that DEM treatment diminished nuclear transport of both fluorescent reporter proteins and endogenous cargos [69, 79, 117]. This is not simply a consequence of stress-induced permeabilization of nuclear envelopes, because the barrier function of nuclear membranes was preserved under these conditions [122]. Since fluorescent reporter proteins like NLS-mCherry or mCit-NES do not contain nuclear or cytoplasmic retention signals, it was reasonable to assume that their stress-induced redistribution reflected changes to the transport apparatus. As described below, such changes were indeed reported by different laboratories, both for severe and mild forms of oxidative stress.

**Figure 4:**
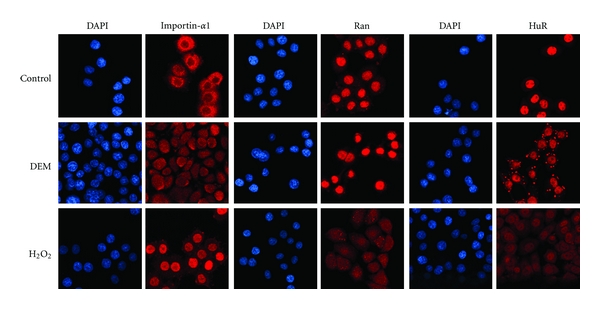
Mild and severe oxidative stress have different effects on nuclear transport factors. The effects of mild (2 mM DEM) and severe oxidative stress (10 mM H_2_O_2_) on the subcellular distribution of importin-*α*1, Ran, and HuR were analyzed in HeLa cells. Proteins were located by indirect immunofluorescence [39, 69, 79]. DEM treatment accumulated importin-*α*1 in nuclei but did not drastically affect the distribution of Ran. By contrast, severe oxidative stress induced by H_2_O_2_ caused a pronounced nuclear accumulation of importin-*α*1 and collapsed the nucleocytoplasmic Ran gradient. Both treatments relocated HuR to the cytoplasm. However, DEM triggered the assembly of HuR-containing SGs, which were rare or absent upon incubation with H_2_O_2_.

A common consequence of H_2_O_2_-induced severe oxidative stress is the collapse of the nucleocytoplasmic Ran GTPase gradient in growing cells (Figure 4); this collapse contributes to classical import inhibition [35, 38, 39, 121]. In addition, three key components of the transport apparatus, nucleoporin Nup153, the carrier importin-*β*1, and importin-*α*1 (Figure 4), redistributed when cells were treated with H_2_O_2_ [39]. Aside from transport factor redistribution, H_2_O_2_ also caused the degradation of Ran, Nup153 and importin-*β*1, both by proteasome and caspase-dependent mechanisms. In addition to growing cells, the consequences of H_2_O_2_ incubation were also examined *in vitro.* In these experiments, oxidant treatment led to a significant reduction of the docking step of nuclear import, as it diminished the binding of importin-*α*1/*β*1/cargo complexes at the nuclear envelope [39].

Our more recent studies investigated how nonlethal oxidative stress affects the transport apparatus. To this end, intracellular glutathione concentrations were depleted with DEM. Unlike severe oxidative stress, DEM incubation caused neither a dissipation of the Ran gradient (Figure 4) nor the degradation of transport receptors. However, DEM treatment mislocalized several transport components, including importin-*α*1, its nuclear exporter CAS as well as nucleoporins Nup153, Nup88, and Nup50 [69]. Nuclear retention was one of the mechanisms that contributed to the oxidant-induced nuclear accumulation of these proteins. Concomitant with nuclear retention, high molecular mass complexes were formed in nuclei that contained importin-*α*1, Nup153, and Nup88. A second mechanism promoting the redistribution of transport factors was the increase in nuclear import for importin-*α*1 and CAS [69]. Notably, the subcellular redistribution of importin-*α*1, CAS, Nup153, and Nup88 was accompanied by changes in their posttranslational modification. For example, DEM augmented the phosphorylation for each factor and increased the *O-*GlcNAc modification of Nup153 [117]. All of these events are possibly linked to oxidant-induced signaling, as the relocation of importin-*α*1, CAS, Nup153 and Nup88 was modulated by MEK → ERK1/2 and PI3K → Akt pathways [117].

Oxidative stress not only inhibits nuclear import, the Crm1 export pathway is sensitive to oxidants as well [79], and our group demonstrated that Crm1-mediated export was inhibited by DEM. Consequently, mCit-NES, a Crm1 cargo predominantly in the cytoplasm of unstressed cells, relocated to nuclei in DEM-treated samples (Figure 2). Several mechanisms participated in the oxidant-induced inhibition of Crm1-dependent export [79]. First, oxidative stress changed the association of Nup358, Nup214, Nup62, and Crm1 with the nuclear envelope and redistributed Nup98. Second, the interaction among these nucleoporins was altered. Third, oxidant treatment impaired Crm1 exit from the nucleus and increased its binding to Ran.

Taken together, these studies revealed that oxidative stress alters several steps of classical nuclear import and export and substantiated the hypothesis that the nuclear transport apparatus is an important target for oxidants. Some of the oxidant-sensitive components are shared by import and export pathways, which might explain why both transport routes are affected in stressed cells.

Work by other groups identified additional transport factors that are likely controlled by ROS homeostasis [72]. For instance, ceramide inhibited nuclear import through a pathway that relied on the MAPK p38 [123]. As ceramide is believed to cause oxidative stress [124, 125], these experiments provide another link between ROS imbalance and changes in nuclear trafficking. This idea is further supported by experiments in smooth muscle cells, where lysophosphatidylcholine modulated RanGAP1 activity [126]. Since lysophosphatidylcholine can induce ROS production [127], RanGAP1 and thereby the generation of RanGDP in the cytoplasm are potential candidates for ROS-dependent regulation. The role of RanGAP1 as an oxidant-sensitive target in the cytoplasm is significant, because RanGAP1 promotes the termination of protein export for all importin-*β* like carriers. Furthermore, RanGAP1 has emerged as target for several MAP kinases [128], emphasizing its potential to serve as a redox-sensitive transport regulator at the NPC.

The idea of redox-dependent control at the nuclear pore is consistent with a recent publication that detected the MAP kinases ERK, p38, and JNK at the NPC [129]. Importantly, all of these kinases are activated and/or redistributed by ROS (Table 1). Moreover Nup50, Nup153, and Nup214 are established ERK targets [130], and their phosphorylation changed several interactions that are important for nuclear transport. Specifically, ERK-dependent modification of Nup50 interfered with its binding to importin-*β* and transportin, which are both carriers of the importin-*β* family. Similarly, when Nup153 and Nup214 were phosphorylated by ERK, their association with importin-*β* was reduced. 

In summary, multiple signaling pathways are activated by oxidants, MAP kinases reside at the NPC or relocate upon stress (see below), and several transport factors are targeted by these kinases. Hence, it is reasonable to propose a simplified chain of events: oxidative stress → signaling → transport factor modification and/or relocation → changes in nuclear trafficking → altered distribution of cargos. This is by no means a one-way street, as nuclear transport factors also play a critical role in modifying signaling events.

An example for the interdependence of signaling and nuclear transport is provided by RanBP3. This transport factor is not only regulated by multiple kinase modules, it also controls signaling [131, 132]. RanBP3 is predominantly located in the nucleus and a binding partner for Ran, RCC1, and Crm1. Aside from participating in Ran translocation to the cytoplasm, RanBP3 may also sequester Ran in the nucleus [131]. Phosphorylation by RSK and Akt can modulate RanBP3 function. In particular, RanBP3 modification is believed to stimulate nuclear import by regulating its interaction with RCC1. In support of this model, nonphosphorylatable mutants of RanBP3 displayed a reduced ability to stimulate RCC1 *in vitro* and caused a partial dissipation of the Ran gradient in growing cells [131]. The emerging scenario is that signaling through Ras → MEK1/2 → ERK1/2 → RSK and PI3 kinase → Akt leads to RanBP3 phosphorylation, thereby maintaining the Ran gradient. Since both signaling pathways are modulated by ROS, it is tempting to speculate that their activation by oxidants will help to preserve or re-establish the Ran gradient in stressed cells.

Besides being a downstream target of several signaling pathways, RanBP3 has a critical role in controlling TGF-*β* signaling [132]. Signaling through TGF-*β* and its receptors have multiple links to oxidative stress [133–136], and many effects of TGF-*β*-like ligands are exerted by the downstream transcriptional regulators Smad2/3. Smad2/3 are shuttling proteins, and their transport to the nucleus relies on direct binding to importin-*β*, without involvement of the adaptor importin-*α* [137]. Following activation of TGF-*β*, Smad2/3 are phosphorylated and accumulate in nuclei, where they regulate the expression of target genes. The termination of TGF-*β* signaling involves the dephosphorylation of Smad2/3 and their export to the cytoplasm. Notably, Smad2/3 nuclear export is not sensitive to leptomycin B, suggesting that Crm1 is not required for exit from the nucleus. Indeed, RanBP3 was identified as a possible carrier that helps to move Smad2/3 to the cytoplasm [132]. Several lines of evidence support this idea; RanBP3 bound nonphosphorylated Smad2/3, interacted with Smad2/3 in the nucleus and promoted Smad2/3 nuclear export in a Ran-dependent fashion. Together, these studies established an essential role for RanBP3 as a negative regulator of Smad2/3 signaling, which relies on its ability to transport Smad2/3 to the cytoplasm.

The impact of ROS on nuclear transport is not limited to signaling-dependent effects, since ROS can directly induce the modification of nuclear transport components. Protein carbonylation is one of the consequences of oxidative stress, and it occurs in an age-dependent fashion for nucleoporins Nup153 and Nup93. Nucleoporin carbonylation correlated with the “leakiness” of NPCs [138], and could be particularly harmful to postmitotic cells, in which some nucleoporins are replaced only slowly. In the context of signaling, it will be interesting to determine whether the age-dependent nucleoporin carbonylation alters the NPC association of MAP kinases or nucleoporin phosphorylation.

In summary, experiments described above suggest that the stress-induced modulation of nuclear trafficking is caused by changes in the concentration, distribution, and posttranslational modification of transport factors [72, 82]. This process is further complicated by the fact that oxidant-dependent relocation of transport factors can be compartmentalized even within the nucleus or cytoplasm, as shown by the formation of cytoplasmic stress granules.

### 2.5. Oxidative Stress, Stress Granule Assembly, and Nuclear Transport

One of the possible consequences of oxidative stress is the formation of cytoplasmic stress granules (SGs). SGs are generated in response to stress that leads to the accumulation of stalled translation initiation complexes [139, 140]. SG assembly is part of a stress defense mechanism that helps to retain and protect mRNAs from degradation. One of the signaling events crucial for the formation of most SGs is Ser51 phosphorylation on eIF2*α* (eukaryotic translation initiation factor 2) [139–141]. Ser51 can be modified by four different upstream kinases, PKR, PERK, GCN2, and HRI (heme-regulated initiation factor 2 kinase), which are activated by various stressors, including the oxidant arsenite. Other signaling events are relevant to SG biogenesis and disassembly; for instance, arsenite promotes the sequestration of Rho and ROCK1 in SGs, possibly to limit the activation of the downstream target JNK [142]. Moreover, focal adhesion kinase (FAK) controls the disassembly of SGs and can be stimulated with H_2_O_2_ [143, 144]. 

In addition to components of the small ribosomal subunit and RNA-binding proteins, arsenite-induced SGs contain importin-*α*1 [145]. Notably, importin-*α*1 knockdown delays SG formation, suggesting a role in the dynamics of SG assembly. These are important data which further substantiate the contribution of nuclear protein transport factors to the stress response. At present, we do not fully understand these events; however, it is conceivable that SGs are one of the “hubs”, where ROS-mediated signaling and nuclear transport components come together in the cytoplasm. Results for the mRNA-binding protein HuR support this idea. HuR shuttles between the nucleus and cytoplasm and relies on importin-*α*1 for nuclear import. Under normal growth conditions, HuR is predominantly in the nucleus, but a 4-hour DEM treatment concentrated HuR in SGs (Figures 3 and 4). At the same time, importin-*α*1 accumulated in nuclei, but it was still detectable in the cytoplasm [69, 117]. It should be emphasized that the association of macromolecules with SGs is dynamic. Proteins as well as RNA can shuttle between SGs and the surrounding cytoplasm [141, 146], and this may also apply to importin-*α*1.

What are the possible mechanisms that promote the ROS-dependent changes in importin-*α*1 and HuR distribution and how are these events linked to SG assembly? The DEM-induced relocation of HuR is likely driven by the combination of importin-*α*1 nuclear accumulation and HuR association with SGs. In particular, concentrating importin-*α*1 in nuclei of stressed cells could diminish nuclear import of HuR. At the same time, importin-*α*1 has a role in SG biogenesis. Although details of this process have yet to be defined, importin-*α*1 may recruit components to cytoplasmic foci that are destined to form SGs. Given that importin-*α*1 binds and transports a variety of cargos, importin-*α*1 shuttling between SG foci and the cytoplasm could accomplish this task. If our model is correct, it could help explain the lack of SG formation in cells incubated with H_2_O_2_ [147, 148]. As shown in Figure 4, H_2_O_2_ did not induce SGs, and importin-*α*1 became highly concentrated in the nucleus, with little of the protein remaining in the cytoplasm. Moreover, stress can also increase nuclear retention and import of importin-*α*1 [113]. As a result of these events, the concentration of importin-*α*1 in the cytoplasm will be low when cells are treated with H_2_O_2_, which in turn could limit the formation of SGs.

The potential contribution of nuclear transport factors to SG assembly or function is not restricted to importin-*α*1. Support for this notion comes from importin-*β* family members importin 8 and transportin which localize to SGs upon arsenite treatment [149, 150]. At this point, we have only few examples that connect nuclear transport components with SGs, and future studies will have to unravel how nuclear trafficking, SG assembly, and ROS-dependent signaling are integrated.

### 2.6. Oxidative Stress and the Subcellular Distribution of Key Signaling Molecules

Elevated levels of ROS modify the activity of redox sensitive components that participate in signaling or other essential biological processes [1, 6, 9, 39, 69, 79, 87, 88, 114, 116, 151–153]. Notably, such ROS-dependent changes in activity are frequently accompanied by the intracellular relocation of the redox-sensitive factors. This scenario applies to a growing list of protein kinases, phosphatases, transcription factors, and components of the nuclear transport apparatus (Table 1). Several of the kinases and phosphatases that redistribute under oxidative stress conditions are key players in signaling circuits, where they control cell survival, growth, proliferation, or death. The interdependence of the activation status and intracellular distribution is crucial for these enzymes, as it determines the specificity and duration of signaling events [152, 154–156]. In the following, we discuss some of the kinases and phosphatases for which oxidant-dependent relocation has been established.

The activity and location of several members of the MAPK and PI3 kinase families are modulated by ROS. Such spatiotemporal control is particularly important for the response to stress, where the repair of stress-induced damage and cell survival relies on the outcome of compartment-specific signaling events. Multiple signaling modules that respond to ROS, both by activation and relocation, have been analyzed in our group [114, 116]. We focused on Akt and ERK1/2, kinases that are essential for signal transduction through PI3 → Akt and MEK → ERK1/2 modules. The stressor DEM elevated the phosphorylation of Akt on Thr308 and Ser473, which leads to Akt activation. At the same time, DEM induced the dual phosphorylation of ERK1/2, thereby activating the MAP kinases. Importantly, DEM not only activates Akt and ERK1/2, but also increased significantly the nuclear/cytoplasmic ratio of phospho-Akt(Ser473) and dually phosphorylated-ERK1/2 [114]. A possible outcome of this shift is a change in the phosphorylation profiles of nuclear and cytoplasmic targets. Notably, the compartmentalization of Akt and ERK1/2-dependent signaling events is even more complex [114], as we demonstrated in the nucleus a direct correlation between the levels of phospho-Akt(Ser473) and phospho-ERK1/2. Our studies suggested that the nuclear concentration of phospho-Akt(Ser473) is dependent on nuclear phospho-ERK1/2 and *vice versa.* Accordingly, crosstalk occurs between phospho-Akt(Ser473) and ERK1/2 in response to oxidative stress; this crosstalk is specific for the nuclear compartment.

More recent work on PI3 kinase by others further emphasizes the importance of the localized action of signaling molecules. The PI3 kinase catalytic subunit p110*β* carries a nuclear localization signal in its C-terminal domain, while the regulatory subunit p85*β* harbors a nuclear export signal. The analysis of a p110*β* transport mutant showed that the ability of the p85*β*/p110*β* complex to regulate cell survival was strictly dependent on its nuclear localization [157]. Although the effect of oxidative stress on the distribution of this kinase has yet to be determined, these findings provide compelling evidence for the control of cell signaling by nuclear transport.

Another example that illustrates the ROS-dependent activation and distribution of protein kinases is the heterotrimeric enzyme 5′-AMP activated kinase (AMPK). AMPK is an energy sensor which plays a pivotal role in the regulation of metabolic homeostasis by phosphorylating targets that are involved in glucose, carbohydrate, lipid, and protein metabolism [158–161]. In unstressed cells, AMPK shuttles between the nucleus and cytoplasm and this shuttling relies on the nuclear exporter Crm1 [116, 162]. However, in response to oxidative stress, AMPK *α*- and *β*-subunits concentrated in the nucleus. This could be accomplished—at least in part—by ROS-induced changes to the nuclear export apparatus, as Crm1 is one of the transport components that are affected by ROS (see above). Interestingly, the link between AMPK activity, subcellular distribution, and nuclear trafficking is even more intricate, as importin-*α*1, a component of the nuclear transport apparatus, is also modified by AMPK [163].

Epidermal growth factor receptor (EGFR) is a receptor tyrosine kinase that is especially important to human health, because signaling through EGFR is linked to tumorigenesis, metastasis and radioresistance. EGFR is located in the plasma membrane, but it also entered the nucleus in response to oxidative stress, heat, or radiation [164]. Moreover, incubation of cultured cells with hydroxy-nonenal, a compound generated by lipid peroxidation, promoted the nuclear accumulation of EGFR [19]. When in the nucleus, EGFR stimulated DNA repair, a process that contributes to radioresistance and potentially limits the success of radiotherapy. Since EGFR is membrane bound, details of its nuclear transport are likely to differ from soluble cargos. Nevertheless, importin-*β*1 and Crm1 (Figure 1) were identified as nuclear carriers that participate in EGFR trafficking [165, 166].

The link between oxidative stress and the localization of key signaling components is not limited to protein kinases. For instance, the lipid and protein phosphatase PTEN has functions in the nucleus and cytoplasm, and oxidative stress promotes PTEN nuclear accumulation [167]. In cells treated with H_2_O_2_, PTEN concentrated in nuclei, where it stabilizes the tumor suppressor p53. Under normal conditions, PTEN is exported from the nucleus by the carrier Crm1 in a cell-cycle dependent fashion, and this export relied on signaling through PI3 kinase [168]. However, incubation with H_2_O_2_ induced PTEN phosphorylation on Ser380, which inhibited its nuclear export [167]. The control of PTEN shuttling upon oxidative stress probably goes beyond the oxidant-induced phosphorylation of the enzyme. As such, the exporter Crm1 is one of the cellular targets that are sensitive to ROS, and signaling through the PI3 kinase → Akt module regulates several components of the nuclear transport apparatus [79, 117]. This interdependence of nuclear transport and signaling is further complicated by the fact that the enzymatic activity of PTEN is regulated by oxidants (see below).

For the examples discussed here, ROS-mediated changes in the nucleocytoplasmic distribution of kinases and phosphatases could reflect the requirement to modify selected substrates in specific subcellular compartments. To this end, the ROS-induced nuclear accumulation of ERK1/2, PI3 kinase, AMPK, EGFR, or PTEN will alter the phosphorylation and activity of nuclear substrates such as transcription factors and other regulators of gene expression. However, such redistribution will also impact other compartments, because the sequestration of kinases or phosphatases in the nucleus can change the phosphoproteome in the cytoplasm as well.

### 2.7. What Is the Interface between the Initial Oxidant Exposure and Changes in the Nuclear Transport Apparatus?

As discussed in previous sections, oxidative stress targets components of the nuclear transport machinery. Moreover, different signaling cascades are implicated in the control of trafficking across the NPC, in part by regulating the posttranslational modification of nuclear transport factors. A complete mechanistic understanding of these events requires that the initial impact of the oxidant can be connected to functional changes of the nuclear transport apparatus. For many of the processes described here, the interface between the primary oxidant-induced event and changes in the posttranslational modification or function of transport factors is not fully defined. In the following, we will, therefore, speculate on some of the possible links.

In principle, two distinct mechanisms can underlie the effect of ROS on nuclear transport factors. First, ROS might react directly with the nuclear transport apparatus, leading to the covalent modification of individual components. Second, oxidative stress could activate signaling cascades that ultimately trigger the phosphorylation and/or *O*-GlcNAc glycosylation of the transport machinery. In the second scenario, signaling begins with a redox-sensitive target that induces a chain of events which conclude with the posttranslational modification of one or more nuclear transport factors.

**Direct Modification of the Nuclear Transport Apparatus by ROS**

In line with what is known about redox-sensitive residues in proteins, we expect that for nuclear transport components cysteine, methionine, lysine, arginine, and histidine residues are among the side chains that are particularly prone to direct oxidation or other ROS-dependent modifications [169]. This idea is supported by a study describing the S-nitrosylation of Crm1 on two cysteine residues and the concomitant inhibition of Crm1-mediated nuclear export [170]. Besides Crm1, nucleoporins are other candidates for a direct modification by ROS or compounds generated upon oxidative stress. Our hypothesis is supported by the increase in nucleoporin carbonylation when cells encounter oxidative stress [138].

**Signaling as Possible Interface between Oxidant Exposure and Nuclear Transport Modification**

Although many of the enzymes that mediate the posttranslational modification of transport factors are known, upstream events regulating these enzymes are less well understood. This applies in particular to the first step of the process, that is, the impact of ROS on its primary target. We propose that protein kinases, phosphatases, or small GTPases that are redox-sensitive [171–174] could fill this gap, as they activate signaling pathways that culminate in transport factor modification. A particularly interesting candidate in this respect is the protein kinase Src, which contains a cysteine switch that is oxidized in order to achieve full kinase activation. Moreover, the redox-dependent stimulation of Src promotes the ligand-independent transphosphorylation of EGFR and subsequent activation of PI3 and ERK kinases [175]. In line with this order of events, it is possible that the ROS-induced formation of disulfide bonds in Src will stimulate the PI3 and ERK-dependent effects on nuclear transport factors as they are discussed here.

The same reasoning applies to several phosphatases [174], including PTEN and low molecular weight protein tyrosine phosphatase (LMW-PTP). PTEN is crucial for the downregulation of PI3 kinase signaling. However, oxidant-induced thiol modification of PTEN inactivates the phosphatase, and thereby promotes signaling through the PI3 kinase → Akt module [174]. With respect to nuclear transport, ROS-induced PTEN inactivation would increase the impact of PI3 kinase on trafficking. In a similar fashion, the redox-dependent inactivation of LMW-PTP leads to sustained ERK activation [176]. This could elevate the ERK-dependent phosphorylation of soluble transport factors and nucleoporins, thus altering their function.

Aside from phosphorylation, *O*-GlcNAc glycosylation of nucleoporins is induced by oxidative stress. The oxidant-dependent increase in *O-*GlcNAc modification is possibly achieved by the complex regulation of *O*-GlcNAc transferase and *β*-*N*-acetylglucosaminidase. At present, these events are not fully understood [177].

Taken together, we propose that changes in the cellular redox homeostasis impact nucleocytoplasmic trafficking by two general mechanisms that are likely to operate in parallel. First, ROS or ROS-generated compounds directly modify redox-sensitive transport factors, this can alter their function. Second, the impact of ROS on redox-sensitive signaling proteins will ultimately modulate the posttranslational modification and activity of nuclear transport components.

### 2.8. Antioxidant Defenses Occur in a Compartmentalized Fashion

In addition to the compartmentalized activation and action of kinases and phosphatases, components of the antioxidant defense apparatus are also unequally distributed within the cell [63, 178]. This is illustrated by catalase, an enzyme concentrated in peroxisomes, and the different forms of superoxide dismutase (SOD) [64, 65, 179, 180]. While manganese-containing SOD (MnSOD) is in the mitochondrial matrix, copper- and zinc-containing SOD (Cu,ZnSOD) can be found preferentially in the cytoplasm and extracellular SOD (EC-SOD) on the cell surface. Moreover, the unequal distribution of GSH and enzymes involved in GSH metabolism will also contribute to subcellular differences in the response to ROS [59, 181–183]. Aside from these enzymes and antioxidants, the localized action of chaperones, critical factors for the repair of stress-induced damage, is well established [115, 184–186]. Since chaperone function is essential for proper signaling and also required for nuclear transport, the nucleocytoplasmic localization and function of heat shock proteins and other chaperones will have significant impact when cells experience ROS imbalances.

We propose that the unequal distribution of antioxidant defense and repair components will impact both cargos and transport factors in a compartment-specific fashion. Accordingly, the prevention and repair of oxidant-induced damage will be different in the nucleus and cytoplasm. Depending on its subcellular location, this could have differential effects on the movement and function of a shuttling protein. For example, nuclear cargos that encounter higher levels of ROS in the cytoplasm could be immobilized in this compartment. The same model can be applied to nuclear transport factors. Thus, nucleoporins on the nuclear and cytoplasmic side of the NPC could be exposed to different levels of ROS and repair. Since nuclear and cytoplasmic nucleoporins participate in different steps of trafficking, damage on either side of the nuclear pore could have unique consequences for nuclear transport.

## 3. Conclusions

The impact of ROS on human health is well established, and links between oxidative stress, nuclear transport, and disease have been defined. For instance, oxidative stress plays a pivotal role in the hyperglycemia-induced damage of multiple tissues and organs [47, 49, 51–53, 55, 187]. GAPDH nucleocytoplasmic shuttling not only participates in these processes, but has also been connected to cancer and neurodegenerative disorders, such as ALS, Alzheimer, or Parkinson disease [88]. Hence, it is conceivable that the oxidant-induced relocation of GAPDH is common to diabetes, cancer, and some forms of neurodegeneration. This shared feature can be extended to the stress-induced nuclear trafficking of the transcriptional regulators NF-*κ*B and Nrf2 and may include other diseases, such as Friedreich ataxia [56, 188, 189].

The examples highlight how the compartment-specific action of signaling molecules, defense and repair reactions provide sophisticated tools to regulate cell physiology. Thus, confining these processes to specific locations will limit the access to downstream targets and clients. In the context of this review, the nucleocytoplasmic distribution of kinases, phosphatases, and other factors involved in posttranslational modification or folding can be expected to directly affect the communication between cytoplasmic and nuclear compartments. This is emphasized by the fact that many of the nuclear transport components and their cargos are modified in an ROS-dependent fashion by phosphorylation, *O*-GlcNAc glycosylation, acetylation, or sumoylation. 

**Figure 5:**
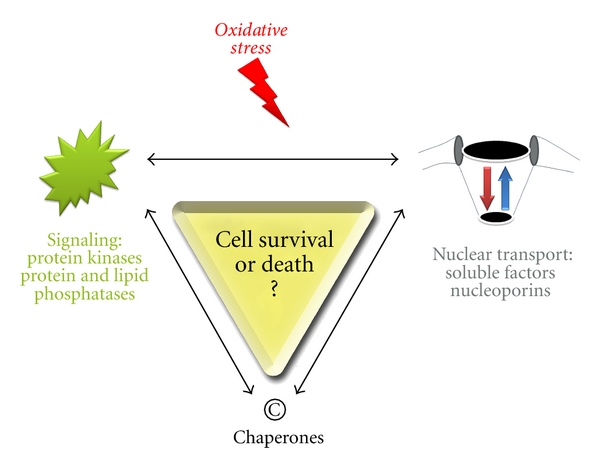
Simplified model for the crosstalk between signaling and nuclear transport in response to oxidative stress. Oxidative stress impinges on signaling molecules and the nuclear transport apparatus, with chaperones modulating both processes. Different scenarios can explain the communication between nuclear transport and signaling pathways in oxidant-treated cells. In one case, oxidative stress alters the localization and activity of transport factors. This will change the subcellular distribution of key signaling molecules, which in turn affects the modification of downstream targets. Alternatively, the signaling pathways activated by oxidative stress cause the modification and redistribution of transport factors. Both scenarios are likely to take place side-by-side, and the balance of these events will ultimately determine cell fate.

Our current understanding of ROS, signaling, and nucleocytoplasmic transport supports the notion that these processes are intricately connected. Although many of the details are still to be discovered, the findings from different fields can be merged into a simplified model. Here, we propose that crosstalk and feedback between different components of this signaling circuit will determine how cells respond to oxidative stress (Figure 5). In one scenario, the activation of signaling pathways promotes the posttranslational modification of nuclear transport factors. This triggers the redistribution of transport factors and alters the movement of cargo across the nuclear envelope. Alternatively, oxidant-induced damage to the transport apparatus could modulate the nucleocytoplasmic localization of kinases or phosphatases, thereby changing the spatiotemporal pattern of signaling. We believe that the two scenarios will take place side by side, affecting different signaling modules and targets in the nucleus and cytoplasm. Both scenarios are further shaped by the localized action of chaperones, which impact both signaling and nuclear transport. The input from signaling, trafficking, and repair will culminate in the decision on cell survival or death. 

As outlined in this review, the dynamic organization of signaling cascades and the nuclear transport apparatus are ideal to respond to internal and external cues. In this context, nucleocytoplasmic trafficking provides the switch to direct events to the nucleus or cytoplasm. The interdependence of signaling and transport pathways provides the flexibility to adjust to a wide variety of changes in cell physiology.

## Figures and Tables

**Table 1:** Redox-sensitive cellular targets in eukaryotic cells. Components that alter their activity and/or nucleocytoplasmic distribution when ROS concentrations increase are listed. See text for details.

Component or process	Effect of ROS
*Signaling proteins, transcriptional regulators*	
JNK, MAPK	Activation
p38, MAPK	Activation, nuclear translocation
ERK1/2, MAPK	Activation, nuclear accumulation
PI3 kinase (some isoforms)	Activation, changes in nucleocytoplasmic distribution
5′-AMP activated kinase	Inhibition, nuclear accumulation; possibly by reduced nuclear export *via* Crm1
Human insulin receptor kinase activity	Activation
Src family kinases	Activation
EGFR	Nuclear translocation; DNA repair
Protein tyrosine phosphatases	Inactivation
PTEN	Nuclear accumulation; association with p53
STAT3	Nuclear translocation
NF-*κ*B, transcription factor	Nuclear accumulation; transcription
FoxO transcription factors	Nuclear translocation (i.e., FOXO1, FOXO3a, and FOXO4)
yAP-1, yeast transcription factor	Nuclear translocation
Msn2p, Msn4p, yeast transcription factors	Nuclear translocation, transcription
CREB	Phosphorylation, nuclear translocation
Nrf2	Nuclear accumulation
HMGB1	Cytoplasmic translocation
HuR, RNA-binding protein	Relocation to cytoplasm, accumulation in stress granules

*Nuclear transport apparatus*	
Classical nuclear import	Inhibition
Crm1-dependent nuclear export	Inhibition
Ran, small GTPase; Gsp1 in *S. cerevisiae *	Relocation to cytoplasm upon severe oxidative stress
Importin-*α*1, adaptor for classical nuclear import	Accumulation in nuclei, accumulation in cytoplasmic stress granules
Crm1, nuclear exporter	Accumulation at nuclear envelope
CAS, exporter for importin-*α*	Nuclear accumulation
Multiple nucleoporins located at different positions within the nuclear pore complex: Nup358, Nup214, Nup88, Nup62, Nup153, Nup50, Nup98, and others	Changes in the association with nuclear envelope; altered nucleocytoplasmic distribution; degradation upon severe stress, in some cases mediated by caspases.
